# Acute Biomechanical Effects of Cardiac Contractility Modulation in Living Myocardial Slices from End-Stage Heart Failure Patients

**DOI:** 10.3390/bioengineering12020174

**Published:** 2025-02-12

**Authors:** Mark F. A. Bierhuizen, Jorik H. Amesz, Sanne J. J. Langmuur, Bobby Lam, Paul Knops, Kevin M. Veen, Olivier C. Manintveld, Jolanda Kluin, Natasja M. S. de Groot, Yannick J. H. J. Taverne

**Affiliations:** 1 Department of Cardiology, Erasmus University Medical Center, 3015 GD Rotterdam, The Netherlands; m.bierhuizen@erasmusmc.nl (M.F.A.B.);; 2 Translational Cardiothoracic Surgery Research Lab, Department of Cardiothoracic Surgery Erasmus University Medical Center, 3015 GD Rotterdam, The Netherlands

**Keywords:** living myocardial slices, cardiac contractility modulation, heart failure, device therapy, translational research

## Abstract

Proof-of-concept to determine the direct biomechanical effects of cardiac contractility modulation (CCM) on living myocardial slices (LMS) from patients with end-stage heart failure (HF). Left ventricular LMS from patients with end-stage HF were produced and cultured in a biomimetic system with mechanical loading and electrical stimulation. CCM stimulation (80 mA, 40 ms delay, 21 ms duration) enhanced maximum contractile force (CCM: 1229 µN (587–2658) vs. baseline: 1066 µN (529–2128), *p* = 0.05) and area under the contractile curve (CCM: 297 (151–562) vs. baseline: 243 (129–464), *p* = 0.05) but did not significantly impact contractile duration, time to peak, or time to relaxation. Increasing CCM stimulation delay, duration, and amplitude resulted in a higher fraction of LMS with a positive inotropic response. Furthermore, CCM attenuated the negative force-frequency relationship in HF-LMS. CCM stimulation enhanced contractile force in HF-LMS. The fraction of LMS exerting a positive inotropic response to CCM increased with increasing delay, duration, and amplitude settings, suggesting that personalizing stimulation parameters could optimize the beneficial effects of CCM. CCM is a novel device-based therapy that may improve contractile function, ejection fraction, functional outcomes, and quality of life in patients with heart failure. However, continuous efforts are needed to identify true responders to CCM therapy, understand the exact mechanisms, and optimize the contractile response to CCM stimulation. The present study revealed that CCM enhanced the contractile force of HF-LMS in a stimulation setting-dependent manner, reaching a larger fraction of the myocardium while increasing delay, duration, and amplitude. This understanding may contribute to the individualization of CCM stimulation settings.

## 1. Introduction

Cardiac contractility modulation (CCM) is a novel device-based therapy for patients with refractory symptomatic heart failure (HF) [1]. CCM stimulation enhances myocardial contractility by delivering non-excitatory electrical pulses of high voltage and long duration to the failing myocardium during the absolute refractory period [2,3]. As such, CCM does not elicit a new contraction but provides inotropic enhancement of contractility by increasing systolic levels of intracellular calcium [4,5]. CCM stimulation of the right ventricular septum improved global contractility, ejection fraction, functional outcomes, and quality of life and elicited reverse remodeling similar to cardiac resynchronization therapy in HF patients [1,6]. However, more studies are necessary to determine whether CCM improves long-term outcomes, such as survival and HF hospitalizations [7,8]. Continuous efforts are needed to identify true responders to CCM therapy, understand the exact mechanisms, and optimize the contractile response to CCM stimulation [9]. Recent studies in 3D human-engineered cardiomyocytes and neuro-cardiac coculture indicated that CCM stimulation settings may impact cardiomyocyte contractile improvement, but these models still lack mechanical loading and physiological resemblance [10,11]. In the current study, living myocardial slices (LMS) from end-stage HF patients were used to evaluate the acute impact of CCM stimulation parameters on the biomechanical properties of the human failing myocardium.

## 2. Methods

### 2.1. Slice Preparation

Left ventricular tissue was obtained from patients with end-stage HF undergoing assist device implantation or cardiac transplantation. All patients consented to the use of their surgical residual material for scientific research (opt-out method of consent), as approved by the Medical Ethical Committee of the Erasmus Medical Center (MEC 2020-0988) and in accordance with local regulations and with the ethical standards as laid down in the 1964 Declaration of Helsinki and its later amendments and guidelines. LMS production was described previously in detail [12,13]. In short, left ventricular biopsies originating from the apex and left ventricular free wall were immediately submerged in 4 °C Tyrode slicing buffer (NaCl 136 mM, KCl 5.4 mM, MgCl_2_·6H_2_O 1 mM, NaH_2_PO_4_·H_2_O 0.33 mM, Glucose 10 mM, CaCl_2_·2H_2_O 0.9 mM, 2,3-butanedione monoxime 30 mM, HEPES 5 mM, pH 7.4) and trimmed to remove epicardial fat and excessive endocardial trabeculae. The biopsies were then immersed in 37 °C 4% low-melting agarose (Agarose II, VWR Chemicals LLC, Solon, OH, USA) and cooled until the gel solidified. Submerged in a 4 °C Tyrode buffer-filled bath, the embedded tissue was sliced by a high-precision cutting vibratome (VT1200S, Leica BioSystems, Nussloch, Germany) with a thickness of 300 µm (vibration amplitude 1.3 mm, blade advance speed 0.07 mm/s). After removal of the agarose, plastic triangles were glued to both ends of the LMS, with longitudinal myocardial fiber orientation in between the triangles.

### 2.2. Slice Cultivation

The cultivation chambers (InVitroSys GmbH, Munich, Germany) were filled with 2.4 mL of 37 °C culture medium (Gibco Medium-199 (Grand Island, NY, USA) supplemented with 5% penicillin-streptomycin, 5% insulin–transferrin–selenium-X, and 50 µM 2-mercaptoethanol). Culture medium calcium concentration was 1.80 mM. From the onset of cultivation, 1.6 mL of medium was refreshed after 1 h and hereafter every 24 h. The slices were mechanically loaded with a diastolic preload of approximately 1 mN using a custom-made stretcher, of which one end was able to move during contraction, resulting in auxotonic contractions [13]. The preload was readjusted with every medium change. The cultivation chambers were placed in a 37 °C 5% CO_2_ incubator and placed on a rocking plate (30 rpm) for continuous oxygen and nutrient supply to the LMS. LMS were continuously cultured with electrical field stimulation using a biphasic square wave main pulse (50 mA), consisting of 3 ms charging and discharging pulses separated by a 1 ms pause interval (total pulse duration: 7 ms). LMS were continuously stimulated at 30 bpm during the first hour to optimize oxygen and nutrient supply to the tissue and with 60 bpm hereafter to simulate near-physiological conditions.

### 2.3. CCM Stimulation

**Figure 1:**
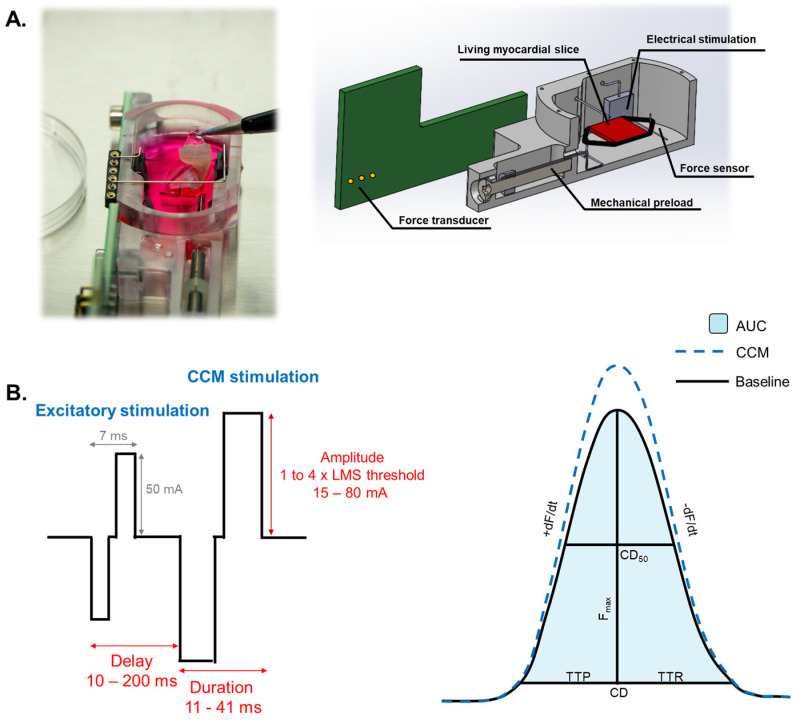
(**A**) illustrates the LMS cultivation set-up. (**B**) shows definitions of excitatory and CCM pulse parameters and contractile measurements. AUC: area under the curve; CD: contraction duration; CD_50_: peak width at 50% of the maximum amplitude; F_max_: maximum contraction force; TTP: time to peak; TTR: time to relaxation; +dF/dt: steepest positive slope; −dF/dt: steepest negative slope.

Clinically, characteristic CCM stimulation involves an amplitude of 7.5 V, delivered as 2 (1–3) biphasic pulses over a 20.56 (10.28–30.84) ms duration, with a delay of 20–45 ms following local sensing. On day one of LMS culture, near-clinical CCM field stimulation was administered as a second biphasic pulse (Figure 1). CCM stimulation was performed with a 40 ms delay after the excitatory stimulus, an 80 mA amplitude, and a total pulse duration of 20 ms to simulate clinical settings. Next, different CCM stimulation parameters (delay, amplitude, and duration) were tested, as specified in Figure 1. The stimulation threshold was determined by increasing baseline pulse amplitude in steps of 5 mA until contractile capture. The effect of CCM pulse amplitude (1 to 4 times the individual LMS stimulation threshold), pulse duration (11, 21, 31, and 41 ms), and pulse delay (10, 20, 30, 40, 50, 70, 100, 150, and 200 ms) was evaluated. Each experiment consisted of 5 min of continuous CCM stimulation, followed by a 15 min recovery period before starting the next experiment.

### 2.4. Functional Refractory Period

For each LMS, the functional refractory period (FRP) was determined using decremental pacing with a fixed S1 rate and decreasing the programmed extra S2 stimuli. In this context, S1 (50 mA) refers to a series of regular electrical stimuli delivered at a fixed interval (1000 ms) to establish a fixed rate, while S2 is a single electrical stimulus (80 mA) delivered at a progressively shorter interval (950–100 ms) after the last S1 stimulus. The first S1–S2 interval that did not show contractile capture on the S2 stimulus was defined as the FRP.

### 2.5. Force-Frequency Relationship

The force-frequency relationship (FFR) was determined by increasing stimulation frequencies from 30 to 180 bpm with increments of 30 bpm and 1 min in between intervals. A comparison was made of the FFR between baseline and during CCM stimulation to assess the effect of CCM at higher stimulation frequencies.

### 2.6. Contractile Measurements

Contractile measurements were extracted using the peak analysis module of LabChart 8 software (ADInstruments). Start and end of the peak were chosen at 10% away from the baseline to compensate for baseline noise [14]. Contractile force was continuously measured by a magnetic force transducer in the cultivation chamber. Contractility was assessed for 60 s directly before each protocol and during the fifth minute of CCM stimulation. For each contraction, force amplitude (F_max_), peak area (AUC), contraction duration (CD), contraction duration at 50% of the maximum amplitude (CD_50_), time to peak (TTP), time to relaxation (TTR), steepest positive slope (+dF/dt), and steepest negative slope (−dF/dt) were evaluated (Figure 1). A positive inotropic response was defined as a ≥5% increase in F_max_ compared to baseline, and a negative response as a ≥5% decrease. A neutral response was defined as a response in Fmax between −5% and 5%. LMS with a positive inotropic response were selected for biomechanical effect analyses. A separate biomechanical analysis was performed in LMS with a positive inotropic response. LMS were excluded from analysis for the respective protocols if baseline noise or sensor errors interfered with the contractile signal.

### 2.7. Statistical Analysis

Statistical analyses were performed using R software (version 4.4.0; R Foundation for Statistical Computing, Vienna, Austria) with the “lmerTest” statistical package. Continuous baseline data were presented as mean ± standard deviation (SD) if normally distributed and as the median and interquartile range (IQR) otherwise. Categorical data were presented as numbers (%). All contractile parameters were presented as median (IQR), as the majority of these data have a non-normal distribution. Differences in contractile parameters were assessed using a clustered Wilcoxon signed rank test. Relative values of biomechanical parameters were calculated as $\frac{\left(CCM\;value-baseline\;value\right)}{baseline\;value}\cdot 100\%$. A linear mixed-effect model was developed with random slopes for stimulation frequency and nested random intercepts for LMS in individual patients to capture higher correlations within patients and the repeated measures within LMS. Fixed effects used were the application of CCM, stimulation frequency, and their interaction term. The model was fit using under restricted maximum likelihood with the Nelder–Mead optimizer [15]. T-tests using Satterthwaite’s method were employed to obtain *p*-values of the fixed effects [16]. *p*-values were considered statistically significant if *p* ≤ 0.05.

## 3. Results

### 3.1. LMS Characteristics

Baseline characteristics of the study population are summarized in Table 1. No patients were previously treated with CCM therapy. LMS were produced from left ventricular biopsies of seven patients with end-stage HF (age: 39 ± 18.5 years, three male). Overall, two LMS showed minimal contractions and were excluded, resulting in 64 LMS. The median FRP of HF-LMS was 275 (260–330) ms. No HF-LMS had an FRP below 200 ms, and no extra induced contractions were observed during CCM stimulation.

### 3.2. Biomechanical Effects of CCM on Contractility

The biomechanical effects of CCM are shown in Table 2. CCM stimulation (80 mA, 21 ms duration, 40 ms delay) significantly enhanced F_max_ in HF-LMS (n = 60) compared to baseline (CCM: 1229 (587–2658) vs. baseline: 1066 (529–2128) µN, *p* = 0.05). Maximum +dF/dt increased significantly with CCM stimulation (CCM: 10,145 (5086–22,260) vs. baseline: 8148 (4109–16,488) µN/s, *p* = 0.05) and –dF/dt (CCM: −6968 (−16,660–−3692) vs. baseline: 6461 (−13,355–−3020) µN/s, *p* = 0.04). In addition, an increased AUC was observed (CCM: 297 (151–562) vs. baseline: 243 (129–464) µN.s, *p* = 0.05). CD, TTP, and TTR were not affected by CCM.

**Figure 2:**
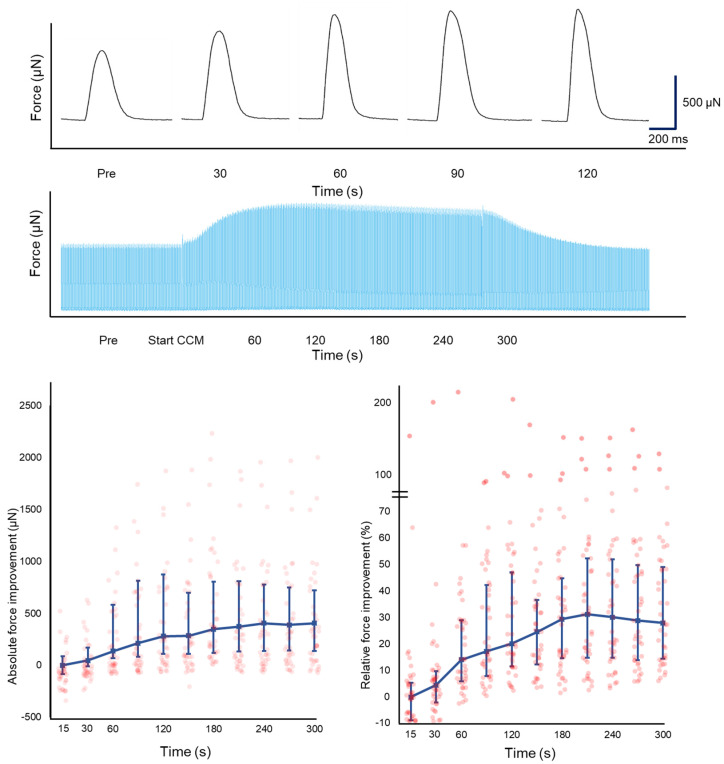
CCM stimulation enhanced F_max_ gradually for up to 3 min and then stabilized in LMS with positive inotropy (n = 42) from heart failure patients (n = 7). Typical contractile tracings were provided. Data are expressed as median and IQR, with added individual data points.

Variation in F_max_ was observed between LMS in response to CCM, even within different LMS produced from the same patient. A total of 42 LMS (70.0%) had a positive inotropic response to CCM, 11 LMS (18.3%) showed a negative inotropic response to CCM, and 7 LMS (11.7%) had a neutral response. In LMS with a positive inotropic response (n = 42), CCM increased F_max_ by 19.5% (CCM: 1324 (801–2738) vs. baseline: 1066 (626–2113) µN, *p* = 0.03), while enhancing AUC (CCM: 306 (192–581) vs. baseline: 243 (156–461) µN.s, *p* = 0.04) and reducing CD_50_ (CCM: 217 (202–242) vs. baseline: 224 (216–252) ms, *p* = 0.04). These results are presented in Table 3 and Supplementary Table S5. In LMS with a positive inotropic response, F_max_ increased gradually for up to 3 min and then stabilized (Figure 2), which disappeared after cessation of CCM stimulation.

Variation in Fmax was also observed between patients in response to CCM stimulation (Table 4). Patients with ischemic cardiomyopathy (n = 3) demonstrated an increase of Fmax (68.4, 35.2, and 5.5%), while both patients with chemo-induced dilated cardiomyopathy responded with a decrease (−0.3 and −4.2%). In both patients, greater CCM delay and duration and, to a lesser extent, amplitude resulted in a maximal increase of Fmax to 34.5 and 14.5%, respectively.

### 3.3. Effect of CCM Stimulation Delay

**Figure 3:**
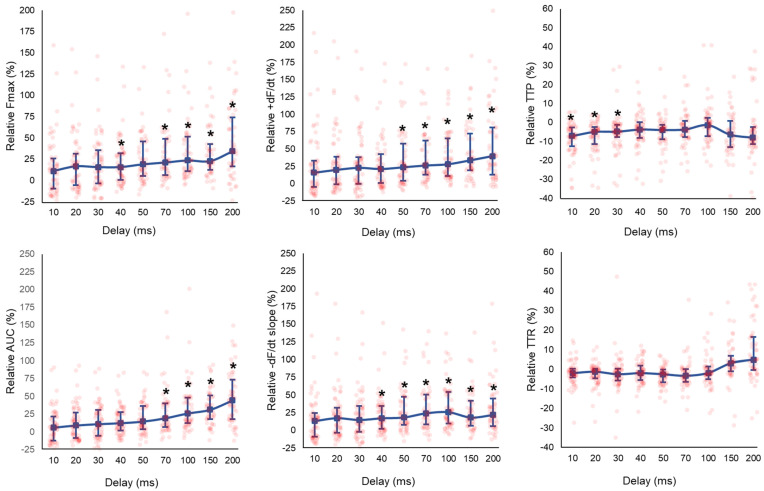
Biomechanical effects of increasing CCM pulse delay (10–200 ms) in LMS (CCM amplitude 80 mA, 21 ms duration). Increasing delay enhanced F_max_, AUC, +dF/dt, and −dF/dt, but did not alter TTP or TTR. Data are expressed as median and IQR, with added individual data points. Clustered Wilcoxon signed rank tests were used to compare contractile parameters for delay 10 ms (n = 60), 20 ms (n = 60), 30 ms (n = 60), 40 ms (n = 60), 50 ms (n = 55), 70 ms (n = 49), 100 ms (n = 55), 150 ms (n = 47), and 200 ms (n = 53). * *p* ≤ 0.05.

LMS displayed enhanced F_max_ in a delay-dependent manner, as illustrated in Figure 3 and Supplementary Table S1. The AUC, dF/dt, and -dF/dt were enhanced with increasing delay settings (Figure 3). A reduction in TTP was observed at delay ≤ 30 ms (delay 30 ms; TTP; CCM: 158 (138–176) vs. baseline: 170 (159–185), *p* = 0.05).

**Figure 4:**
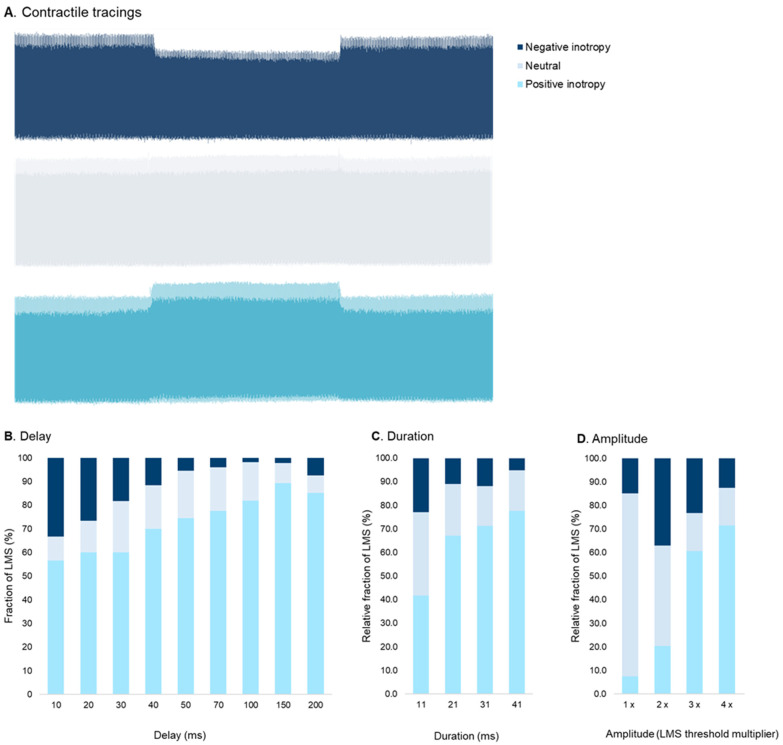
(**A**): Contractile tracings of HF-LMS with a negative, neutral, and positive inotropic response during CCM stimulation. (**B**–**D**)*:* Inotropic response of HF-LMS to varying CCM stimulation parameters. A higher fraction of LMS showed a positive inotropic response with increasing CCM pulse delay, amplitude, and duration. Fractions were calculated for pulse delay 10 (n = 60), 20 (n = 60), 30 (n = 60), 40 (n = 60), 50 (n = 55), 70 (n = 49), 100 (n = 55), 150 (n = 47), and 200 ms (n = 53); pulse duration 11 (n = 48), 21 (n = 64), 31 (n = 59), and 41 ms (n = 58); pulse amplitude 1× (n = 54), 2× (n = 54), 3× (n = 56), and 4× threshold (n = 56).

The fraction of LMS exerting a positive inotropic response to CCM increased with increasing delay settings (Figure 4). At the shortest delay of 10 ms, 34 LMS (57%) demonstrated a positive inotropic response, which increased to ≥70% of LMS with delays ≥ 40 ms. Of note, 33% of LMS demonstrated a negative inotropic response at a delay of 10 ms. This fraction subsided with increasing delay settings (Figure 4).

### 3.4. Effect of CCM Stimulation Duration

Longer stimulation duration showed increased F_max_ (Supplementary Table S2). Stimulation duration of 11 ms did not significantly increase F_max_, and only 20 LMS (41%) demonstrated a positive inotropic response at this duration. The fraction of LMS with a positive inotropic response increased to 78% with a 41 ms stimulation duration (Figure 4).

### 3.5. Effect of CCM Stimulation Amplitude

The fraction of LMS with a positive inotropic response increased from 4 LMS (7%) at one times threshold to 40 LMS (71%) at four times threshold (Figure 4). The effect of CCM stimulation amplitude on median F_max_ is demonstrated in Supplementary Table S3.

### 3.6. Force-Frequency Relationship

**Figure 5:**
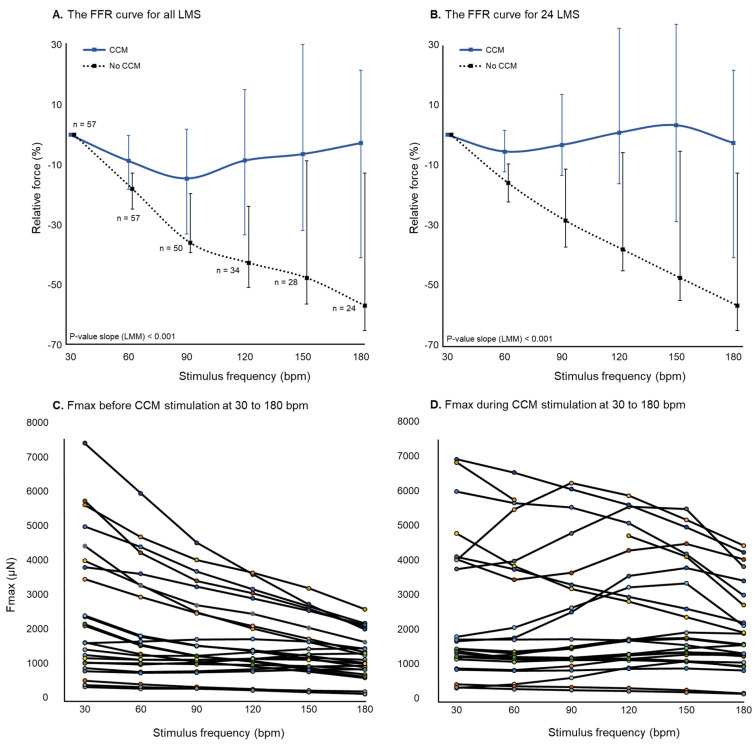
(**A**): Partial reversal of the negative force-frequency relationship (FFR) with CCM stimulation. The number of included LMS at higher frequencies decreased from 57 to 24 due to exclusion of LMS when contractions did not capture all electrical stimuli. (**B**): The FFR of 24 LMS with capture of all pacing frequencies (30 to 180 bpm). (**C**,**D**) illustrate the F_max_ of 24 LMS before and during CCM stimulation at 30 to 180 bpm, respectively.

HF-LMS predominantly demonstrated a neutral or negative FFR before CCM stimulation, as demonstrated by a decrease in median F_max_ with increasing stimulation frequencies (Figure 5). Positive, neutral, and negative FFR were all observed when applying simultaneous CCM stimulation (Figure 5). A decrease in sample size was observed at higher stimulation frequencies, due to intolerance of HF-LMS to high-frequency stimulation. Panel B of Figure 5 illustrates a similar FFR in 24 LMS with capture at all stimulus frequencies. The results of the linear mixed-effect model are presented in Supplementary Table S4. F_max_ decreases with increasing stimulation frequencies was significantly less steep in LMS with CCM stimulation compared to without CCM stimulation (β CCM: −0.04 vs. no CCM: −0.12, *p*-interaction < 0.001). Residuals of the linear mixed-effect model were normally distributed based on visual inspection.

## 4. Discussion

### 4.1. Key Findings

This is a proof-of-concept study using CCM stimulation in LMS with heart failure phenotype, showing an acute increase in maximum contractile force in the majority of LMS. The strength of response was dependent on stimulation parameters, and the improved biomechanical profile seemed to be stronger in patients with ischemic cardiomyopathy. Increasing CCM pulse delay, duration, and amplitude resulted in a higher fraction of LMS with a positive inotropic response. Significantly higher force generation was observed with increasing delay and duration, but not amplitude. Furthermore, CCM stimulation attenuated the negative force-frequency relationship in the failing myocardium. All biomechanical effects were abolished upon cessation of CCM therapy.

### 4.2. Effect of CCM on the Biomechanical Profile of HF-LMS

In our study, CCM stimulation with near-clinical settings (delay 40 ms, amplitude 80 mA, duration 21 ms) enhanced maximum and total contractile force, and contraction and relaxation slopes in LMS obtained from patients with HF, while CD, TTP and TTR were not affected. In other words, CCM stimulation increases cardiac contractility without altering systolic and diastolic intervals. Similar increased contractile force and slopes were observed in pre-clinical studies [4,10,11,17], and their clinical counterparts demonstrated no significant alterations of echocardiographic ejection times or diastolic filling times [3,18]. In contrast, pre-clinical studies found a small increase in TTP in 3D cardiomyocytes and endocardial trabeculae obtained from explanted failing hearts [4,10]. Model specificity and loading conditions could explain these differences. Illustratively, multiple different platforms to culture LMS have been developed, each more closely resembling physiological loading conditions. The differences in mechanics of each of these approaches can be appreciated on the force–length plane. Auxotonic loading more closely resembles physiological loading conditions compared to unloaded or isometrically loaded conditions [19,20]. In our study, biomechanical properties of HF-LMS were assessed using auxotonic loading, displaying greater physiological relevance in comparison to unloaded cardiomyocytes or isometric loaded trabeculae from failing hearts [13].

### 4.3. Individual Response of LMS to CCM Stimulation

A larger fraction of LMS showed a positive inotropic response when CCM pulse amplitude, duration, and delay increased. These variations were not reported in other pre-clinical studies and might be unique for our model, as different layers of the human myocardium were used. Intrinsic transmural heterogeneity has been demonstrated previously in LMS by Pitoulis et al. [21]. It could also be specific to the failing myocardium. Other pre-clinical studies generally use cardiomyocytes with little cell-to-cell coupling and lacking micro-architectural structures as present in the patient [10,11]. However, Burkhoff et al. demonstrated no individual differences when CCM was applied to intact human trabeculae of patients with end-stage HF (n = 6) [4]. Nevertheless, a subset of positive responders was seen in our study and raises the question of whether this effect could be attributed to the underlying disease. A correlation and stratification according to etiology could not be performed due to the patient sample size and variance in the number of LMS per patient (2–20 LMS); however, patients with ischemic cardiomyopathy were amongst the strongest inotropic responders independent of CCM settings, contrary to the two patients with chemo-induced dilated cardiomyopathy, who seemed to benefit more from a greater delay, amplitude, and duration. Further studies should investigate this potential effect of underlying etiology on CCM response. All things considered, our observations suggest that CCM stimulation should optimally strive to be longer in pulse duration and amplitude in order to target a larger fraction of the myocardium.

Although clinical CCM stimulation has not been associated with ventricular arrhythmias, delay settings must be chosen with care, as high-voltage signals have the potential to induce ventricular depolarization or even arrhythmias when administered outside the absolute refractory period. Clinically, CCM is applied 20–45 ms after local sensing. Our results demonstrate that increasing the delay past 45 ms is beneficial to enhance Fmax and AUC in LMS, corroborating previous reports using cardiomyocytes [10,22]. As such, using the maximal delay settings within current safety regulations may already contribute to reaching a larger proportion of tissue and better contractile performance. The duration of the absolute refractory period of ventricular cardiomyocytes is approximately 200–300 ms [23]. In our study, all HF-LMS had an FRP above 200 ms, and no extra induced contractions were observed during CCM stimulation, suggesting that delay settings could potentially be extended safely beyond 45 ms. Inherently, implications for safety should be explored further before implementation and should be correlated with the individual FRP.

### 4.4. CCM and the Force Frequency Relationship in Failing Myocardium

Increasing stimulation frequencies resulted in a reduction of Fmax in LMS from HF patients. A flat or negative FFR is a characteristic feature of the failing myocardium and is often associated with the deterioration of intracellular calcium handling [24]. In the failing myocardium, downregulated protein expression of the sarcoplasmic reticulum (SR) Ca2+ ATPase 2a (SERCA2a) and increased calcium extrusion by the sodium-calcium exchanger result in lower SR storage capacity [24]. In HF-LMS, a neutral or positive FFR was observed more often when CCM stimulation was applied simultaneously. Although there was no significant difference in FFR with and without CCM stimulation, visualization of the FFR curve is suggestive of preservation of contractility at higher stimulation frequencies. CCM is able to improve SERCA2a calcium affinity by rapid phosphorylation of phospholamban, thus increasing calcium sequestration in the SR [5]. Potentially, improved calcium handling by CCM contributes to a partial reversal of the negative FFR in the failing myocardium. Hashimoto et al. demonstrated that SERCA2a gene transfer in transgenic mice improved calcium handling and twitch force, especially at higher stimulation frequencies, which increased the intracellular calcium load [25]. Detailed calcium imaging is required to elaborate on these findings. Potential improvement of the FFR by CCM might partly explain the improvements in functional parameters, such as peak VO2, 6 min walking distance, and quality of life, that were demonstrated in various clinical trials [1].

### 4.5. Clinical Perspective

CCM stimulation is a promising therapeutic strategy that has been applied in hundreds of patients clinically, yet despite its potential benefits, it remains to be universally established in heart failure management guidelines [8,26]. Heart failure is a heterogeneous condition, and response to CCM therapy can vary greatly depending on factors such as stimulation settings, heart failure etiology, and degree of cardiac dysfunction. CCM aims to enhance myocardial contractility by providing electrical stimulation during the refractory period, which may be more beneficial for certain subgroups of patients. Therefore, studies are warranted to identify which patients would benefit most from CCM. In this context, HF-LMS offers valuable insights as they contain disease phenotypes where the tissue is mechanically loaded and electrically stimulated whilst maintaining (micro)architectural form and function [27,28]. Compared to other experimental models, HF-LMS better resembles heart failure etiology, structure, and physiology. Moreover, LMS can be cultured for weeks, or even months, offering a sustained model for long-term studies.

In our study, we demonstrated an individualized response of HF-LMS to different stimulation settings, an effect that has not been demonstrated in other models. This response variation may be attributed to differences in the underlying etiology of heart failure. Based on these observations, we believe that future research using HF-LMS should focus on: (1) the response to therapy in patients with various underlying etiologies, (2) the determination of optimal stimulation settings, and (3) the effects of chronic stimulation over weeks and months on contractility, therapeutic response, and the force-frequency relationship.

## 5. Limitations

This is a proof-of-concept study focusing on the direct effects of CCM in a novel biomimetic model using human LMS derived from patients undergoing cardiac surgery. The use of human tissue is scarce, resulting in a small sample size, which should be increased in future studies to allow for examination of the effect of the underlying disease. Furthermore, a large variability in LMS biomechanical profiling is present, which is inherent to the use of a dynamic system. Nevertheless, our study clearly shows specific trends when using CCM. Healthy human cardiac tissue is even more scarce, and in our expertise, it has a different biomechanical profile compared to HF-LMS. Therefore, each LMS was used as their own control. Additionally, the CSA of LMS was not systematically measured in this study, which precludes precise normalization of force to tension. While our statistical approach and use of LMS as their own control address inter-sample variability, future studies should incorporate CSA measurements to enhance the robustness of biomechanical analyses. Next, this study investigated the acute effects of CCM stimulation parameters on the contractile performance of HF-LMS but did not investigate optimal combined settings for maximal contractile improvement, nor did we extend to chronic CCM stimulation.

## 6. Conclusions

We present evidence that CCM inotropic response is stimulation parameter dependent (delay, duration, amplitude) using a novel biomimetic set-up with human HF-LMS. Furthermore, CCM can partially mitigate the negative FFR of HF-LMS.

## Figures and Tables

**Table 1:** Baseline characteristics.

Patient Characteristics	N = 7
Age, years	39 ± 18.5
Male, n (%)	3 (43)
Etiology of heart failure	
Ischemic cardiomyopathy, n	3
Dilated cardiomyopathy, n	3
Chemotherapy induced, n	2
Myocarditis, n	1
Arrhythmogenic cardiomyopathy, n	1
Surgery	
LVAD implantation, n	2
Cardiac transplantation, n	5
LVAD in situ, n	1

± Standard deviation.

**Table 2:** Comparison between biomechanical parameters before and during CCM stimulation (80 mA, 21 ms duration, 40 ms delay) in LMS (n = 60) from patients with HF (n = 7).

	Baseline	CCM	*p*-Value
F_max (_µN)	1066 (529–2128)	1229 (587–2658)	0.050 *
CD (ms)	450 (396–485)	429 (377–482)	0.297
CD_50_ (ms)	223 (210–256)	217 (199–246)	0.056
–dF/dt (µN/s)	−6461 (−13,355–−3020)	−6968 (−16,660–−3692)	0.043 *
+dF/dt (µN/s)	8148 (4109–16,488)	10145 (5086–22,260)	0.050 *
AUC (µN.s)	243 (129–464)	297 (151–562)	0.053
TTP (ms)	166 (152–187)	160 (142–185)	0.357
TTR (ms)	270 (230–305)	268 (221–303)	0.388

Data are expressed as median (IQR). Differences in contractile parameters were assessed using a clustered Wilcoxon signed rank test. * *p* ≤ 0.05.

**Table 3:** Comparison between biomechanical parameters before and during CCM stimulation (80 mA, 21 ms duration, 40 ms delay) in LMS from patients with HF with a positive inotropic response (n = 42).

	Baseline	CCM	*p*-Value
F_max (_µN)	1066 (626–2113)	1324 (801–2738)	0.030 *
CD (ms)	441 (393–485)	411 (370–474)	0.163
CD_50_ (ms)	224 (216–252)	217 (202–242)	0.043 *
–dF/dt (µN/s)	−6461 (−13,183–−3828)	−7914 (−16,915–−4505)	0.028 *
+dF/dt (µN/s)	8880 (5419–16,282)	11275 (6629–24,770)	0.027 *
AUC (µN.s)	243 (156–461)	306 (192–582)	0.040 *
TTP (ms)	166 (154–186)	159 (142–173)	0.209
TTR (ms)	267 (228–301)	260 (220–281)	0.272

Data are expressed as median (IQR). Differences in contractile parameters were assessed using a clustered Wilcoxon signed rank test. * *p* ≤ 0.05.

**Table 4:** Patient-specific F_max_ (µN) improvement with CCM stimulation.

Patient etiology	Baseline (µN)	CCM (µN)	%	LMS (n)
Ischemic cardiomyopathy	1411 (1042–2113)	2376 (1239–2918)	68.4	20
Ischemic cardiomyopathy	582 (405–863)	786 (532–1113)	35.2	5
Myocarditis-induced dilated cardiomyopathy	1103 (204–1485)	1164 (317–1928)	12.3	5
Arrhythmogenic cardiomyopathy	2668 (1247–4071)	2927 (1267–4202)	9.7	15
Ischemic cardiomyopathy	1103 (624–2167)	1164 (629–2002)	5.5	8
Chemo-induced dilated cardiomyopathy	267 (161–384)	266 (143–454)	−0.3	11
Chemo-induced dilated cardiomyopathy	2267 (1652–2880)	2172 (1671–2671)	−4.2	2

Data are expressed as median (IQR). Percentages were calculated as (CCM − baseline)/baseline × 100%.

## Data Availability

The raw data supporting the conclusions of this article will be made available by the authors on request.

## Supplementary Materials

The following supporting information can be downloaded at https://www.mdpi.com/article/10.3390/bioengineering12020174/s1, Table S1: F_max_ (µN) with various CCM pulse delay settings with fixed amplitude (80 mA) and pulse duration (21 ms); Table S2: F_max_ (µN) with various CCM pulse duration settings with fixed amplitude (80 mA) and pulse delay (40 ms); Table S3: F_max_ (µN) with various CCM pulse amplitude settings with fixed pulse delay (40 ms) and duration (21 ms); Table S4: The results of the linear mixed model. The slope of the squared root F_max_ decrease with increased stimulation frequencies was significantly less steep in LMS (N = 24) with CCM stimulation compared to without CCM stimulation; Table S5: Etiology, F_max_ (µN) and inotropic response before and during CCM stimulation with pulse amplitude 80 mA, delay 40 ms and duration 21 ms in LMS (n = 60) from patients with end-stage heart failure (n = 7).
