# Supplementary material for: Acute Biomechanical Effects of Cardiac Contractility Modulation in Living Myocardial Slices from End-Stage Heart Failure Patients
Source: Bioengineering (Basel). 2025 Feb 12;12(2):174. doi: 10.3390/bioengineering12020174 (PMC11851609; doi:10.3390/bioengineering12020174)
Supplement: Supplementary file 1 [file bioengineering-12-00174-s001.zip › bioengineering-3407259-supplementary.pdf]

Supplementary material

Acute Biomechanical Effects of Cardiac Contractility Modulation in Living Myocardial Slices From End-Stage Heart Failure Patients

Mark F. A. Bierhuizen [1,2], Jorik H. Amesz [2], Sanne J. J. Langmuur [2], Bobby Lam [1,2], Paul Knops [1,2], Kevin M. Veen [2], Olivier C. Manintveld [1], Jolanda Kluin [2], Natasja M. S. de Groot [1,2] and Yannick J. H. J. Taverne [2,*]

[1] Department of Cardiology, Erasmus University Medical Center, Rotterdam and the Netherlands
[2] Translational Cardiothoracic Surgery Research Lab, Department of Cardiothoracic Surgery Erasmus University Medical Center, Rotterdam, the Netherlands
* Correspondence: y.j.h.j.taverne@erasmusmc.nl

Table S1. F_{max} (μN) with various CCM pulse delay settings with fixed amplitude (80 mA) and pulse duration (21 ms).

Delay	Baseline	CCM	P-value	N
10 ms	1060 (532 – 2223)	1154 (579 - 2553)	0.408	60
20 ms	1038 (542 - 2253)	1185 (628 - 2663)	0.156	60
30 ms	1072 (550 - 2342)	1206 (672 - 2807)	0.140	60
40 ms	1066 (529 - 2128)	1229 (587 - 2658)	0.050*	60
50 ms	1150 (536 - 2325)	1434 (643 - 2977)	0.056	55
70 ms	1167 (391 - 1937)	1307 (569 - 2847)	0.030*	49
100 ms	1294 (452 - 2695)	1625 (729 - 3533)	0.028*	55
150 ms	1345 (461 - 2637)	1603 (706 - 3635)	0.024*	47
200 ms	1392 (669 - 2773)	1794 (908 - 3488)	0.033*	53

Data are expressed as median (IQR). Differences in contractile parameters were assessed using a clustered Wilcoxon Signed Rank test. *$p \leq 0.05$.

Table S2. F_{max} (μN) with various CCM pulse duration settings with fixed amplitude (80 mA) and pulse delay (40 ms).

Duration	Baseline	CCM	P-value	N
11 ms	915 (390 - 2852)	909 (433 - 3075)	0.380	48
21 ms	1163 (543 - 2636)	1383 (650 - 3178)	0.073	64
31 ms	1439 (609 - 2815)	1648 (802 - 3327)	0.076	59
41 ms	1455 (642 - 3049)	1791 (897 - 3546)	0.029*	58

Data are expressed as median (IQR). Differences in contractile parameters were assessed using a clustered Wilcoxon Signed Rank test. *$p \leq 0.05$.

Table S3. F_{max} (μN) with various CCM pulse amplitude settings with fixed pulse delay (40 ms) and duration (21 ms).

Amplitude	Baseline	CCM	P-value	N
1 x threshold	1242 (722 – 2103)	1192 (685 - 2092)	0.056	54
2 x threshold	1189 (675 - 2150)	1178 (672 - 2058)	0.274	54
3 x threshold	1196 (667 - 2177)	1226 (817 - 2396)	0.635	56
4 x threshold	1186 (663 - 2226)	1387 (907 - 2797)	0.151	56

Data are expressed as median (IQR). Differences in contractile parameters were assessed using a clustered Wilcoxon Signed Rank test. *$p \leq 0.05$.

Table S4. The results of the linear mixed model. The slope of the squared root F_{max} decrease with increased stimulation frequencies was significantly less steep in LMS (N = 24) with CCM stimulation compared to without CCM stimulation.

Predictors	Estimates	CI	P-value
Intercept	47.58	36.61 – 58.54	< 0.001*
Stimulation frequency	-0.12	-0.15 – -0.09	< 0.001*
CCM stimulation	-3.37	-5.08 – -1.67	< 0.001*
Stimulation frequency x CCM stimulation	0.08	0.06 – 0.10	< 0.001*

A linear mixed-effect model was developed with random slopes for stimulation frequency and nested random intercepts for LMS in individual patients to capture higher correlations within patients and the repeated measures within LMS. Fixed effects used were the application of CCM, stimulation frequency and their interaction term. The model was fit using under restricted maximum likelihood with the Nelder-Mead optimizer. T-tests using Satterthwaite's method were employed to obtain p-values of the fixed effects. *$p \leq 0.05$.

Table S5. Etiology, F_{max} (μN) and inotropic response before and during CCM stimulation with pulse amplitude 80 mA, delay 40 ms and duration 21 ms in LMS (n = 60) from patients with end-stage heart failure (n = 7).

Patient etiology	Patient	LMS	Baseline (μN)	CCM (μN)	%	Inotropic response
Ischemic cardiomyopathy	Patient 1	1	788.7	1004	27.3	Increase
		2	566.4	750.2	32.5	Increase
		3	938.1	1222	30.3	Increase
		4	243.8	314.6	29.0	Increase
		5	581.5	786.2	35.2	Increase
Ischemic cardiomyopathy	Patient 2	6	1026	1354	32.0	Increase
		7	1627	2778	70.7	Increase
		8	2122	2418	13.9	Increase
		9	1761	4116	133.7	Increase
		10	1195	1399	17.1	Increase
		11	2145	2964	38.2	Increase
		12	397.2	568.7	43.2	Increase
		13	2086	2481	18.9	Increase
		14	797.9	1200	50.4	Increase
		15	2950	4601	56.0	Increase
		16	1117	1175	5.2	Increase
		17	1108	2333	110.6	Increase
		18	106.7	115.1	7.9	Increase
		19	1788	2618	46.4	Increase
		20	1089	1665	52.9	Increase
		21	2564	3408	32.9	Increase
Ischemic cardiomyopathy	Patient 3	22	605.8	592.4	-2.2	Neutral
		23	1040	1294	24.4	Increase
		24	677.5	739.2	9.1	Increase
		25	416.1	504.7	21.3	Increase
		26	2481	2237	-9.8	Decrease
		27	3788	4318	14.0	Increase
		28	1225	1235	0.8	Neutral
		29	1166	1093	-6.3	Decrease
Chemo-induced dilated cardiomyopathy	Patient 4	30	161.1	168.1	4.3	Neutral
		31	153.4	143.3	-6.6	Decrease
		32	116.1	119.3	2.8	Neutral
		33	260.4	242.5	-6.9	Decrease

		34	329.5	348	5.6	Increase
		35	164.3	142.2	-13.5	Decrease
		36	383.9	454.1	18.3	Increase
		37	266.9	266	-0.3	Neutral
		38	342.1	327.7	-4.2	Neutral
		39	829.7	800.7	-3.5	Neutral
		40	695.8	902.9	29.8	Increase
Chemo-induced dilated cardiomyopathy	Patient 5	41	3495	3172	-9.2	Decrease
		42	1038	1171	12.8	Increase
Myocarditis-induced dilated cardiomyopathy	Patient 6	43	204.8	316.8	54.7	Increase
		44	1485	1928	29.8	Increase
		45	799.6	898.3	12.3	Increase
Arrhythmogenic cardiomyopathy	Patient 7	46	1042	1191	14.3	Increase
		47	608.8	846.6	39.1	Increase
		48	1247	1645	31.9	Increase
		49	4455	4202	-5.7	Decrease
		50	1611	1593	-1.1	Neutral
		51	3788	3639	-3.9	Neutral
		52	2910	2927	0.6	Neutral
		53	2464	2599	5.5	Increase
		54	1281	1267	-1.1	Neutral
		55	2668	3503	31.3	Increase
		56	2682	3541	32.0	Increase
		57	4071	6241	53.3	Increase
		58	4088	4732	15.8	Increase
		59	7740	8952	15.7	Increase
		60	313.4	341.4	8.9	Increase
